# Hybrid Offline–Online Configuration Planning Approach for Continuum Robots Based on Real-Time Shape Estimation

**DOI:** 10.3390/s26041129

**Published:** 2026-02-10

**Authors:** Hexiang Yuan, Zhibo Jing, Yibo He, Jianda Han, Juanjuan Zhang

**Affiliations:** 1 College of Artificial Intelligence, Nankai University, Tianjin 300350, China; yuanhexiang@mail.nankai.edu.cn (H.Y.); zhibojing@nankai.edu.cn (Z.J.); heyibo@mail.nankai.edu.cn (Y.H.); hanjianda@nankai.edu.cn (J.H.); 2 Institute of Robotics and Automatic Information Systems, Nankai University, Tianjin 300350, China; 3 Academy for Advanced Interdisciplinary Studies, Nankai University, Tianjin 300350, China

**Keywords:** continuum robot, configuration planning, shape estimation, configuration refinement, collision avoidance

## Abstract

Continuum robots possess highly flexible backbones, enabling remarkable adaptability and dexterity for motion in confined environments. However, this flexibility also introduces significant nonlinearities and uncertainties, making motion planning under physical constraints particularly challenging. To address this, a hybrid offline–online configuration planning framework is proposed in this work. Specifically, the configuration planning problem is formulated as a nonlinear optimization task that considers collision avoidance and structural constraints. A co-evolutionary strategy is incorporated into the differential evolution (DE) algorithm to decompose the target high-dimensional optimization problem. Then, an unscented Kalman filter (UKF)-based strategy is presented for real-time shape estimation using tip pose feedback for safe distance monitoring. Based on this shape feedback, an online configuration refiner is designed to locally adjust the preplanned configurations, thus leveraging the global perspective of the offline planning configuration to steer the continuum manipulator through constrained spaces. Validation and comparative experiments demonstrate the effectiveness of the proposed method, as well as its enhanced motion smoothness and safe motion performance in real-world environments.

## 1. Introduction

Continuum robots, leveraging their high flexibility and passive compliance, exhibit superior adaptability in confined spaces compared to traditional rigid robots. These properties have enabled continuum robots to demonstrate significant advantages in applications such as minimally invasive surgery [1,2], aero-engine maintenance [3,4], in-space inspection [5], and nuclear reactor maintenance [6]. Nevertheless, their inherent high nonlinearity and strong coupling between multiple degrees of freedom pose substantial challenges for planning and executing collision-free motion in constrained environments.

To enable reliable operation of continuum robots in narrow and constrained environments, configuration planning has attracted considerable research attention. A representative approach is the sampling-based planning method, which has been widely adopted for continuum robots due to its probabilistic completeness. Pan et al. [7] reformulated the configuration planning problem of manipulators as a path-planning problem in constrained spaces, using the rapidly exploring random tree (RRT) algorithm to find the initial path and fitting it with a quadratic Bézier curve. Gao et al. [8] developed an improved RRT-based path-planning method for multi-segment continuum robots, which incorporates kinematic constraints to prevent excessive deformation. However, it mainly focuses on applications in two-dimensional environments. Lang et al. [9] proposed an improved RRT-Connect algorithm and obtained a continuous trajectory by interpolating the joint-space parameters. To avoid strong nonlinear effects arising from joint-space planning, Meng et al. [10] proposed a workspace-guided RRT*-based algorithm, where Jacobian-based inverse kinematics is employed to evaluate the reachability between adjacent workspace nodes. In addition, improved strategies combining RRT and inverse kinematics have been proposed to plan collision-free motions for manipulators in constrained spaces [11,12,13]. Despite their ability to probabilistically guarantee the discovery of a feasible solution within finite time, the inherent random sampling nature makes it difficult to obtain smooth and globally optimal configuration sequences.

Optimization-based methods have demonstrated effectiveness in continuum robot motion planning and shown advantages in balancing multiple performance criteria. Qin et al. [14] developed an optimization-based pat- planning method for hyper-redundant space robots, in which viewpoints are continuously optimized while ensuring collision avoidance. Wang et al. [15] integrated a particle swarm optimization (PSO) algorithm to optimize the random sampling process in path planning and designed constraint units to limit deflection angles of the target path. Inverse-kinematics-based (IK-based) optimization methods [16,17] determine discrete-time configurations by minimizing end-effector pose deviation while satisfying constraints; however, they are typically unable to perform simultaneous planning of configurations and tip trajectory. Yang et al. [18] transformed the motion planning problem of continuum robots into an optimization framework subject to collision avoidance and input constraints. Niu et al. [19] proposed a forward search algorithm with region clipping to optimize manipulator configurations in constrained environments. Wu et al. [20] formulated the manipulator configuration planning for one step as a backbone-path-based optimization problem for collision avoidance. However, most optimization-based planning methods mainly focus on the isolated configuration at one time step, while being limited in the simultaneous planning of full configuration sequences and motion paths.

Additionally, the backbone is typically assumed to have uniform and constant curvature. This simplification makes it difficult to account for significant configuration deviations caused by uncertainty in real-world environments, thereby potentially leading to unsafe motion, especially in densely obstacle-constrained environments. Although the studies in [21,22] present collision avoidance control methods enhanced by real-time configuration adjustment, the generation of target tip trajectory is not addressed. In response to these challenges, this work proposes a hybrid offline-online configuration planning framework based on real-time shape estimation to enable collision-free motion for continuum robots in constrained spaces. Within this framework, a UKF-based estimation strategy is developed to enable real-time shape sensing based on tip pose feedback, providing accurate shape information and distance perception that serve as critical inputs for online collision avoidance. Additionally, unlike most existing methods, the continuum manipulator is guided by a globally optimized configuration sequence, thereby leveraging the global perspective of full configurations to facilitate passability and collision avoidance in constrained environments. The main contributions of this work are summarized as follows:

1. A configuration pre-planning method based on the co-evolutionary improved DE (CoDE) is proposed, where each flexible segment is modeled as a sub-population sharing elite solutions to enhance exploration and overall planning quality.
2. A UKF-based real-time shape estimation strategy is presented to reconstruct the manipulator’s shape under friction and deformation effects, enabling reliable feedback for configuration refinement and collision avoidance.
3. An online refiner is designed within the hybrid framework for local configuration adjustment, leveraging the global perspective advantage of offline planning for guidance and achieving improved collision avoidance performance.
4. Validation and comparative experiments in different constrained environments demonstrate its effectiveness and performance benefits in continuum robot configuration planning tasks.

## 2. Kinematic Modeling for Continuum Robot

**Figure 1:**
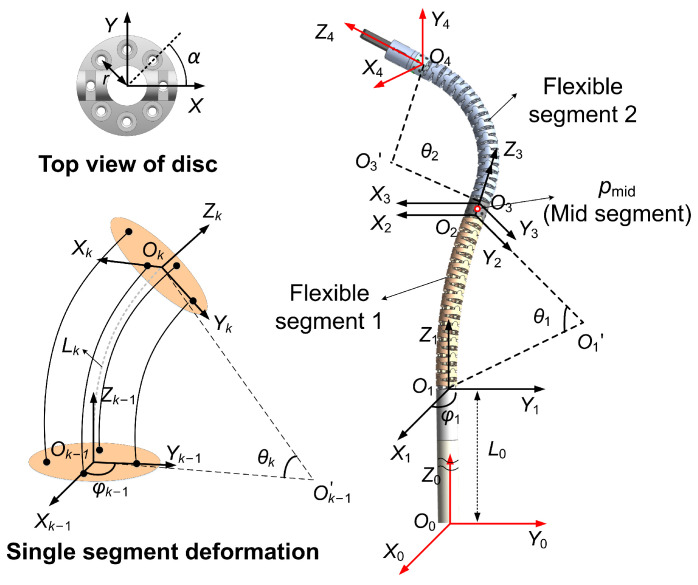
Schematic and coordinate frame definitions of the manipulator.

The geometric configuration and coordinate definition of the continuum manipulator are shown in Figure 1. For any non-singular configuration, the deformation of a single segment of the continuum manipulator can be characterized by its bending angle $\theta_{i}$ in the plane $O_{k-1}O_{k-1}^{\prime }O_{k}$ and its azimuth $\varphi_{i}$ about the $Z_{k-1}$-axis. The overall shape of the segment can be approximated as a constant-curvature arc [23,24,25].

For a single segment, the mapping between its configuration parameters and the tip’s position in the local coordinate frame can be expressed as

(1) pk  k−1=Lkθkcφk−cθkcφksφk−cθksφksθk

where $L_{k}$ denotes the length of the *k*-th flexible segment, c(·) is the abbreviation for cos(·), and s(·) represents the abbreviation for sin(·).

Therefore, the position of the manipulator’s tip in the base frame $O_{0}$ is given by

(2) p2=fw,cqc=P·T3243T21T10Tpo41

where $q_{c}={\left[\theta_{1},\varphi_{1},\theta_{2},\varphi_{2},z_{T}\right]}^{T}\in R^{5}$ is the configuration parameter for the robot. $P=\left[\begin{array}{cc} I_{3} & 0 \end{array}\right]\in R^{3\times 4}$ is a dimensional transformation matrix, and Tk  k−1 is the homogeneous transformation matrix between adjacent frames, which is given by

(3) Tk  k−1=Rk−1pk  k−101×31∈SE(3)

(4) Rk  k−1=RotZk−1,φk·RotYk−1,θk·RotZk,−φk

Likewise, the position of the mid-segment in the base frame can be derived as

(5) pmid=hw,cqc=P·T1021T·pmidTo2,1T=0.5Lmidcφ1sθ1−cφ1cθ1−1L1/θ10.5Lmidsφ1sθ1−sφ1cθ1−1L1/θ1L0+zT+0.5Lmidcθ1+sθ1L1/θ1

The direction of the manipulator’s tip can be expressed as

(6) v2=R3243R21R10R·v^

where $\hat{v}\in R^{3}$ is the direction vector of the tip in the local frame $O_{4}$.

The actuation parameters are defined as $q_{A}={\left[q_{1,1},q_{1,2},q_{2,1},q_{2,2},z_{T}\right]}^{T}\in R^{5}$, where $q_{k,i}$ denotes the deformation length of the *i*-th tendon in the *k*-th flexible segment. Its relationship with the configuration parameters can be derived as follows [26]:

(7) $$q_{k,i}=L_{k}-\theta_{k}rc_{\varphi_{k}+\left(i-1\right)\alpha }$$

where *r* is the radial distance from the tendon to the center of the vertebral joint (Figure 1).

By inverting (7), the mapping between the configuration parameters and the actuation parameters can be obtained:

(8) $$q_{c}=f_{c,A}\left(q_{A}\right)$$

Furthermore, by substituting (8) into (2), the mapping between the tip position and the actuation parameters is obtained:

(9) $$p_{2}=f_{w,A}\left(\cdot \right)=f_{w,c}\left(f_{c,A}\left(q_{A}\right)\right)$$

## 3. Co-Evolutionary DE-Based Offline Configuration Planning

To achieve reliable collision avoidance and smooth motion in constrained spaces, a CoDE-based configuration pre-planning algorithm for the continuum manipulator is developed in this section. The configurations of the two flexible segments are evolved as independent populations, with information exchanged via an elite pool to enhance the algorithm’s exploitation of the search space. This offline planning process aims to provide reference configurations for online refinement in the real world, guiding the manipulator to reach the desired position around a globally optimized configuration sequence.

### 3.1. Continuization of Sparse Key Configurations

**Figure 2:**
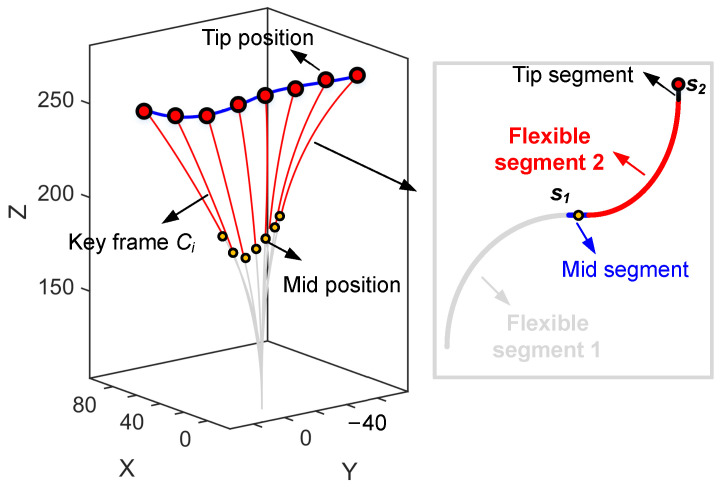
Sequence of key configuration frames.

As shown in Figure 2, a sequence of key configuration frames is defined as $C=\left\{C_{1},C_{2},\ldots ,C_{N}\right\}$, where each key frame $C_{i}$ is parameterized by $C_{i}=\left(\theta_{1,i},\varphi_{1,i},\theta_{2,i},\varphi_{2,i},z_{T_{,i}}\right)$. The motion trajectory of the manipulator body is obtained by continuously interpolating these sparse configurations based on the trajectories of the tip and the mid-segment.

For each key frame, the corresponding mid-segment position and tip position are computed using (5) and (2), respectively. To balance smoothness and computational efficiency, cubic spline interpolation is adopted to smooth the trajectory defined by the tip positions of the key frames. The overall configuration of the manipulator is jointly determined by the mid-position (denoted as $s_{1}$ in Figure 2) and the tip position (denoted as $s_{2}$ in Figure 2). Therefore, the manipulator’s configuration can be fully described by these two key positions. For any given position pair $\left(s_{1},s_{2}\right)$, the corresponding configuration parameters $C_{i}$ can be obtained through inverse kinematics. Furthermore, uniform discretization of the mid-segment and tip trajectories yields the complete configuration sequence corresponding to the current set of key frames.

#### 3.1.1. Segment 1 Configuration via Mid-Segment Position

As shown in Figure 1, for any non-singular configuration, the azimuth angle corresponding to the mid-segment position $p_{\mathrm{mid}}=\left(p_{\mathrm{mid},x},p_{\mathrm{mid},y},p_{\mathrm{mid},z}\right)$ is given by

(10) $$\varphi_{1}=\arctan\frac{p_{\mathrm{mid},y}}{p_{\mathrm{mid},x}}$$

Substituting $k=\frac{p_{\mathrm{mid},x}}{c_{\varphi_{1}}}$ into (5) yields

(11) $$\frac{c_{\theta_{1}}-1}{0.5L_{\mathrm{mid}}s_{\theta_{1}}-k}=\frac{s_{\theta_{1}}}{p_{\mathrm{mid},z}-\left(L_{0}+z_{T}+0.5L_{\mathrm{mid}}c_{\theta_{1}}\right)}$$

Setting $t=\tan\frac{\theta_{1}}{2}$ and then transforming (11) as follows:

(12) $$\frac{\left(p_{\mathrm{mid},z}-L_{0}-z_{T}+0.5L_{\mathrm{mid}}\right)\left(1-t^{2}\right)}{1+t^{2}}+\frac{2kt}{1+t}=0.5L_{\mathrm{mid}}-L_{0}+p_{\mathrm{mid},z}-z_{T}$$

By combining (10) and (12), $\theta_{1}$ can be derived as

(13) $$\theta_{1}=2\arctan\frac{p_{\mathrm{mid}\;,x}}{\cos\left(\arctan\frac{p_{\mathrm{mid}\;,y}}{p_{\mathrm{mid}\;,x}}\right)\left(0.5L_{\mathrm{mid}\;}-L_{0}+p_{\mathrm{mid}\;,z}-z_{T}\right)}\in \left(-\pi ,\pi \right]$$

**Figure 3:**
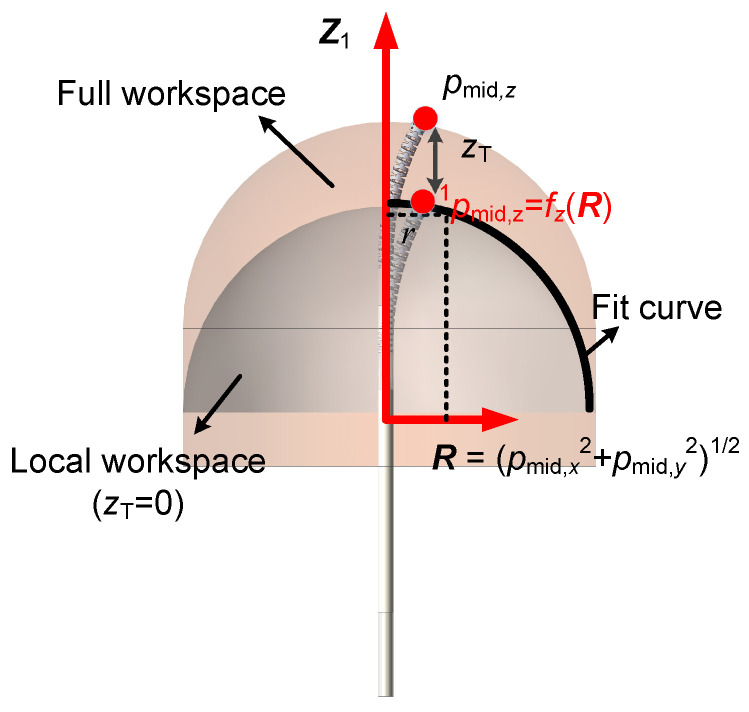
Geometric configuration of flexible segment 1.

As shown in Figure 3, when $z_{T}=0$, a mapping relationship exists between $p_{\mathrm{mid},z}$ and the radial distance *R*. By using (5), the corresponding parameter $p_{\mathrm{mid},z}$ and $R=\sqrt{p_{\mathrm{mid},x}^{2}+p_{\mathrm{mid},y}^{2}}$ for different $\theta_{1}$ under the condition $z_{T}=0$ are calculated to construct the fitting dataset. Based on the fifth-order Gaussian model, the following mapping relationship can be obtained:

(14) $$p_{\mathrm{mid},z}=f_{z}\left(R\right)=\sum_{i=1}^{5}a_{i}e^{\left(-\frac{{\left(R-b_{i}\right)}^{2}}{2c_{i}^{2}}\right)}$$

where $a_{i}$, $b_{i}$, and $c_{i}$ are model parameters identified by the least squares method.

Therefore, configuration parameters at position $p_{\mathrm{mid}}$ can be determined by the following equation:

(15) $$q_{C}^{\left(1\right)}=g_{J,w}\left(p_{\mathrm{mid}}\right)={\left[\theta_{1}\left(\cdot \right),\varphi_{1}\left(\cdot \right),f_{z}\left(\sqrt{p_{\mathrm{mid},x}^{2}+p_{\mathrm{mid},y}^{2}}\right)-p_{\mathrm{mid},z}\right]}^{T}$$

where $g_{J,w}\left(\cdot \right)$ is the kinematic mapping from the workspace to the configuration space.

#### 3.1.2. Segment 2 Configuration via Tip Position

The configuration of the second flexible segment can be derived using differential inverse kinematics based on the tip position, together with the known configuration of the first segment obtained from (15). By reducing the dimensionality of the variable space, the overall manipulator configuration can be computed efficiently.

Given the current configuration parameters $q_{2,k}=\left[\theta_{2,k},\varphi_{2,k}\right]\in R^{2}$ of the second flexible segment, the configuration $q_{2,k+1}$ at the next time step is given by

(16) $$q_{2,k+1}=q_{2,k}+\alpha J_{2}^{-1}\frac{{\left(p_{d}-f_{w,c}\left(q_{c,k}\right)\right)}_{1:2}^{T}}{∥{\left(p_{d}-f_{w,c}\left(q_{c,k}\right)\right)}_{1:2}∥}$$

where $q_{c,k}={\left[\theta_{1},\varphi_{1},q_{2,k},0\right]}^{T}$, and the local Jacobian matrix $J_{2}=\left[\frac{\partial f_{w,c}\left(\cdot \right)}{\partial \theta_{2}},\frac{\partial f_{w,c}\left(\cdot \right)}{\partial \varphi_{2}}\right]\in R^{2\times 2}$. $p_{d}\in R^{3}$ is the desired position of the manipulator’s tip in the workspace. ${\left(\cdot \right)}_{1:N}$ denotes the first *N* dimensions of the current vector. α is the step size coefficient.

Based on the initial configuration $q_{2,k}$, the algorithm iteratively approaches the target configuration via (16). At each iteration, the nonlinear kinematics are locally linearized around the current configuration. The specific procedure is shown in Algorithm 1.

In Algorithm 1, the overall inverse kinematics problem is reduced to a two-dimensional subspace for the solution. Since the local Jacobian matrix $J_{2}$ is square, each iteration involves only several basic matrix operations, thus resulting in low computational overhead. Experimental validation shows that on a personal computer equipped with an Intel^®^ Core^TM^ Ultra 5 225H 1.70 GHz processor, the computation process can typically be completed within 0.1 ms, with a deviation below 0.5 mm.
**Algorithm 1** Configuration for the Second Flexible Segment1:**Input:** Desired position $p_{d}$, current configuration $q_{2,k}$, tolerance λ, step size α, maximum iteration *K*.2:**Output:** Configuration parameter $q_{c}^{\left(2\right)}\in R^{2}$ for the second flexible segment.3:Calculate current tip position: $p_{2}=f_{w,c}\left(q_{2,k}\right)$.4:Calculate current error: $e=p_{d}-p_{2}$.5:Initialization k←0.6:**while** ∥e∥>λ and k<K **do**7:   Calculate local Jacobian matrix $J_{2}$.8:   Calculate new $q_{2,k}$ by (16).9:   Update $p_{2}$ and *e*.10:   k←k+1.11:**end while**12:Set $q_{c}^{\left(2\right)}=q_{2,k}$.

Furthermore, by sequentially computing each discrete position pair $\left(s_{1},s_{2}\right)$, the complete configuration sequence corresponding to the current key frame can be obtained as $q_{s}=\left\{C_{1},C_{2},\ldots ,C_{M}\right\}$.

### 3.2. Evaluation of Configuration Planning Solution

In the proposed framework, the objective of offline planning is to guide a continuum robot from its current configuration to a designated operational region within a constrained space without collision. To this end, the trajectory of the end-effector should be as short and smooth as possible, while maintaining safe separation from surrounding constraint regions over the entire motion process.

For a given configuration sequence $q_{s}$, the total length of the manipulator tip’s motion trajectory can be expressed as

(17) $$f_{L}=\sum_{k=1}^{N_{s}-1}∥f_{w,c}\left(q_{s,k+1}\right)-f_{w,c}\left(q_{s,k}\right)∥$$

where $N_{s}$ denotes the total number of configurations in the sequence set $q_{s}$, and $q_{s,k+1}$ represents the *k*-th configuration within $q_{s}$.

To achieve a smooth end-effector trajectory, the key configuration frames should avoid overly dense clustering in local regions of the workspace. A metric quantifying this distribution uniformity can be defined as

(18) $$f_{\eta }=\sum_{k=1}^{2}\frac{\sigma_{k}}{\mu_{k}}$$

(19) $$\begin{array}{cc} \; & \mu_{k}=\frac{1}{N-1}\sum_{i=1}^{N-1}∥f_{w,c}\left(q_{s,k+1}\right)-f_{w,c}\left(q_{s,k}\right)∥\\ \; & \sigma_{k}=\sqrt{\frac{1}{N-1}\sum_{i=1}^{N-1}{\left(∥f_{w,c}\left(q_{s,k+1}\right)-f_{w,c}\left(q_{s,k}\right)∥-\mu_{k}\right)}^{2}} \end{array}$$

where *N* is the number of optimized key frames. $\mu_{1}$ and $\sigma_{1}$ represent the mean and standard deviation of the mid-position distances across these key frames, while $\mu_{2}$ and $\sigma_{2}$ denote the corresponding mean and standard deviation of the tip position distances.

Furthermore, the configuration planning problem of a multi-segment continuum manipulator can be formulated as

(20) $$\begin{array}{cc} \min_{q_{s}^{\prime }} & g\left(q_{s}^{\prime }\right)=f_{L}+\omega_{\eta }f_{\eta }+\omega_{a}\sum_{i=1}^{N-2}g_{a}\left(q_{s,i},q_{s,i+1},q_{s,i+2}\right)+\sum_{i=1}^{N_{\mathrm{obs}\;}}P_{i}\\ \;s.t.\; & \theta_{i}\in \left(0,\frac{\pi }{2}\right],\varphi_{i}\in \left[-\pi ,\pi \right) \end{array}$$

where $\omega_{\eta }$ and $\omega_{a}$ are two weighting coefficients, $q_{s}^{\prime }\in R^{N\times 5}$ represents the set of optimized key frames, and $g_{a}\left(q_{s,i},q_{s,i+1},q_{s,i+2}\right)$ denotes the angle of the path segment formed by the mid-positions of three adjacent manipulator configurations. The hard constraint term corresponds to the mechanical limits of the manipulator structure, ensuring that all deformations remain within the feasible operating range.

Given the minimum allowable clearance ε between the manipulator and constraint regions, the penalty term $P_{i}$ is defined as

(21) $$P_{i}=\left\{\begin{array}{cc} P & \rho \left(C_{i}\right)\leq R_{obs}+d/2+\varepsilon \\ 0 & \;\mathrm{otherwise}\; \end{array}\right.$$

where $\rho \left(C_{i}\right)$ denotes the minimum distance between the manipulator and the constraint center under configuration $C_{i}$, and *d* is the outer diameter of the manipulator.

### 3.3. Co-Evolutionary Improved DE Algorithm for Configuration Planning

The two-segment continuum manipulator exhibits a highly nonlinear and multimodal mapping between its configuration space (C-Space) parameters and tip positions. To improve exploration of the solution space and facilitate the discovery of feasible configuration sequences, a co-evolutionary strategy is introduced for DE to decompose the high-dimensional nonlinear optimization problem.

#### 3.3.1. Standard DE Algorithm

Given the robustness of the DE algorithm for complex nonlinear optimization [27,28], it is adopted to solve the planning problem of (20), with further enhancements to improve its exploration capability. The core procedure consists of four main steps:

(1) Initialization operation: Initialize the population by randomly generating Np individuals within the predefined parameter search space:
  (22) $$x_{i}^{\left(0\right)}=\left(x_{i,1},x_{i,2},\ldots ,x_{i,D}\right),\;i=1,2,\ldots ,Np$$
  where the value for each parameter dimension is generated by uniform random sampling within its defined domain.
(2) Mutation operation: For the target individual $x_{i}^{\left(g\right)}$ in the *g*-th generation, apply the differential mutation strategy to generate the corresponding mutant vector $v_{i}^{\left(g+1\right)}$:
  (23) $$v_{i}^{\left(g+1\right)}=x_{r1}^{\left(g\right)}+F\cdot \left(x_{r2}^{\left(g\right)}-x_{r3}^{\left(g\right)}\right)$$
  where $r_{1}$, $r_{2}$, and $r_{3}$ are distinct randomly selected individual indices; *F* is the scaling factor controlling the differential perturbation magnitude.
(3) Crossover operation: Recombine the target individual $x_{i}^{\left(g\right)}$ and the mutant individual $v_{i}^{\left(g+1\right)}$ with a certain probability to generate the trial one $u_{i}^{\left(g+1\right)}$:
  (24) $$u_{i,j}^{\left(g+1\right)}=\left\{\begin{array}{cc} v_{i,j}^{\left(g+1\right)}, & \;\mathrm{if}\;\mathrm{rand}\left(0,1\right)\leq CR\;\mathrm{or}\;j=j_{\mathrm{rand}\;}\\ x_{i,j}^{\left(g\right)}, & \;\mathrm{otherwise}\; \end{array}\right.$$
  where CR is the crossover probability, *j* denotes the index of the current individual in the population, and $j_{rand}$ is a randomly selected dimension index.
(4) Selection operation: Select new individual between $x_{i}^{\left(g\right)}$ and $u_{i}^{\left(g+1\right)}$ based on the fitness value obtained by (20):
  (25) $$x_{i}^{\left(g+1\right)}=\left\{\begin{array}{cc} u_{i}^{\left(g+1\right)}, & \;\mathrm{if}\;g\left(u_{i}^{\left(g+1\right)}\right)\leq g\left(x_{i}^{\left(g\right)}\right)\\ x_{i}^{\left(g\right)}, & \;\mathrm{otherwise}\; \end{array}\right.$$

#### 3.3.2. Co-Evolutionary Strategy

To enhance the exploration capability in the configuration space, a co-evolutionary strategy is introduced into the standard DE framework. Specifically, the configuration parameters of each flexible segment are treated as independent sub-populations, thereby decomposing the original high-dimensional decision variables and enabling parallel co-evolution of the configuration parameters corresponding to different segments. The sub-populations share a common pool of elite individuals, periodically exchanging high-quality solutions to facilitate the transfer of configuration parameters between segments, thereby enhancing the algorithm’s exploration capability in the solution space.

**Figure 4:**
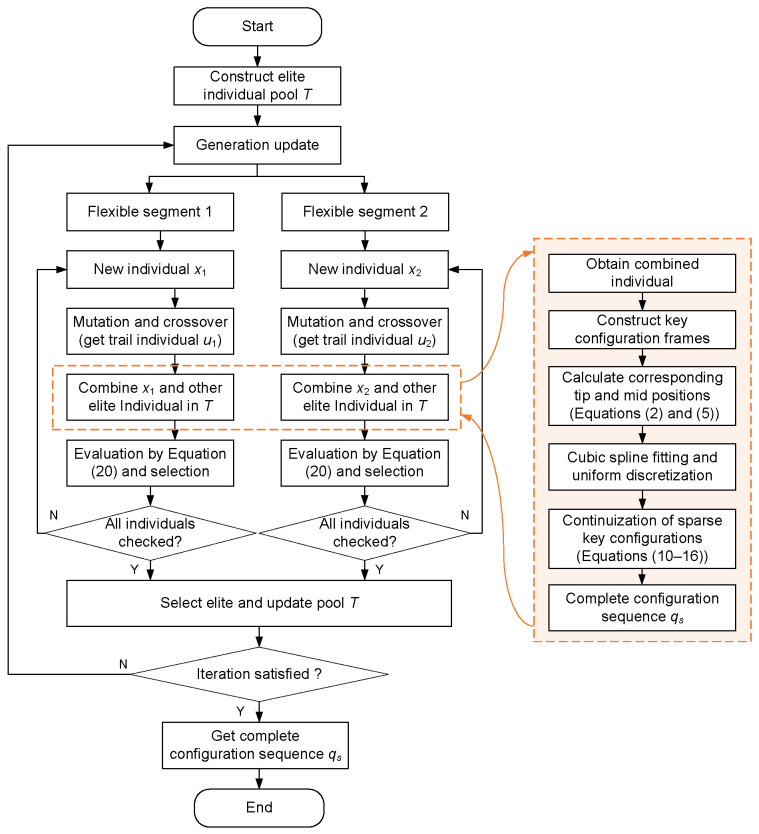
Framework of the CoDE algorithm.

**Figure 5:**
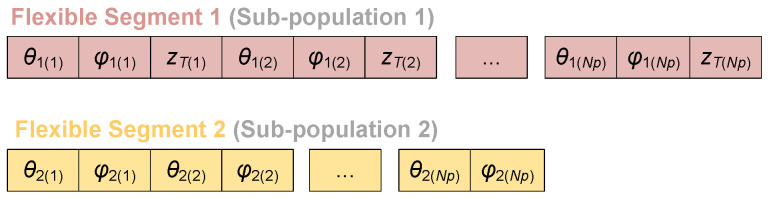
Encoding scheme for configuration parameters.

Figure 4 illustrates the overall framework of the CoDE algorithm. First, each sub-population is initialized independently, and one individual is randomly selected from each to form the representative individual pool *T*. The size of this pool remains constant and equals the number of sub-populations. Subsequently, each sub-population undergoes an independent evolutionary process. In the present case, the two sub-populations correspond to the configuration parameters of two distinct flexible segments, with their encoding scheme shown in Figure 5. During evolution, mutation, crossover, and selection operations are performed sequentially on individuals within a sub-population. The resulting trial individual is then combined with the corresponding representative individual extracted from pool *T*. The quality of this combined solution is evaluated based on the fitness function given in (20). This procedure iterates until all individuals in the sub-population have been evaluated. Finally, the individual yielding the optimal combined fitness is selected as a new elite individual to update the pool *T*, replacing the corresponding elite.

## 4. UKF-Based Real-Time Shape Estimation

To enable real-time perception of the manipulator’s shape and monitor its relative position with respect to the surrounding environment, a UKF-based shape estimation strategy is presented in this section.

Due to friction at the vertebral joints and cumulative friction along the driving tendons, the manipulator experiences shape loss in both the bending and azimuthal directions. To model this effect, the Euler curve is introduced to describe curvature attenuation in bending [29], assuming a linear variation in curvature along the arc length. The variation in the manipulator’s curvature leads to motion lag at its tip. For the *k*-th flexible segment, the bending angle along the arc length *s* of the backbone can be expressed as

(26) $$\theta_{k}\left(\eta ,s\right)=\int_{0}^{s}\left(\eta_{k}s+\frac{{\bar{\theta }}_{k}}{L_{k}}\right)ds$$

where $\eta_{k}$ represents the time-varying curvature damping factor in the bending direction, and ${\bar{\theta }}_{k}$ is the bending angle under the ideal constant-curvature condition.

Given the relatively small attenuation of deformation in the azimuthal direction, a time-varying damping coefficient σ is introduced to provide a linear approximation of the manipulator’s azimuthal deformation characteristics:

(27) $$\varphi_{k}\left(\sigma ,s\right)={\bar{\varphi }}_{k}-\sigma_{k}s$$

where $\varphi_{k}\left(\sigma ,s\right)$ is the azimuth angle at the arc length *s*, and ${\bar{\varphi }}_{k}$ is the azimuth angle under the ideal constant-curvature condition.

Therefore, for the *k*-th flexible segment, the position along the backbone at arc length *s* can be derived as

(28) pO2k−1s,ψ,qc=∫sθiηi,k,scφiσi,k,sds,∫sθiηi,k,ssφiσi,k,sds,∫cθiηi,k,sdsT

where $\psi =\left[\eta_{1},\sigma_{1},\eta_{2},\sigma_{2}\right]$ is the time-varying damping factor vector.

Furthermore, the position of any point on the manipulator in the base frame $O_{0}$ is given by

(29) p^ks,ψ,qc=∏i=12k−1Ti  i−1·P·pO2i−1s,ψk,qc

Substituting (26) and (27) into (4), the tip’s direction can be derived as

(30) v^ks,ψ,qc=∏i=14Ri  i−1(ψ)·v^

where $\hat{v}$ is the direction vector of the tip in the local frame $O_{4}$.

The state vector of the curvature damping factor at the *k*-th time step is defined as $\psi_{k}={\left[\eta_{1,k},\sigma_{1,k},\eta_{2,k},\sigma_{2,k}\right]}^{T}$. Given the slow time-varying nature of $\psi_{k}$, the following random walk model is used:

(31) $$\psi_{k+1}=\psi_{k}+\omega_{k}$$

where $\omega_{k}∼N\left(0,Q_{\mathrm{UKF}}\right)$ is a zero-mean white Gaussian noise, and $Q_{\mathrm{UKF}}\in R^{4\times 4}$ is the process noise covariance matrix.

The position and orientation of the manipulator tip are taken as the measurement vector, denoted as $z_{k}=\left[{\hat{p}}_{k,\;\mathrm{tip}\;}\;{\hat{v}}_{k,\;\mathrm{tip}\;}\right]\in R^{6}$. The measurement model at the *k*-th time instant is given by

(32) $$z_{k}=h\left(\psi_{k},q_{c}\right)+\zeta_{k}$$

where $h\left(\psi_{k},q_{c}\right)=\left[\hat{p}\left(\tilde{L},\psi_{k},q_{c}\right)\;\;\hat{v}\left(\tilde{L},\psi_{k},q_{c}\right)\right]$ is the observation function, $\tilde{L}$ is the manipulator’s total length, and $\zeta_{k}∼N\left(0,R_{\mathrm{UKF}}\right)$ is the zero-mean white Gaussian measurement noise with measurement noise covariance matrix $R_{\mathrm{UKF}}\in R^{6\times 6}$.

Considering the nonlinear nature of the observation model, the standard six-step UKF algorithm [30] is employed to estimate the state vector $\psi_{k}$. Equation (29) allows the spatial position at any arc length *s* along the manipulator to be efficiently calculated, which enables real-time shape reconstruction of the entire manipulator.

## 5. Online Refinement of Manipulator Configuration

Due to inherent passive compliance and nonlinear friction effects, multi-segment continuum manipulators exhibit significant uncertainties, making offline-planned configuration solutions insufficient for reliable motion in practical constrained environments. Therefore, an online re-planner is designed for configuration refinement and collision avoidance based on model predictive control (MPC). The online planning process is guided by the pre-planned configurations, thereby exploiting the global insight of offline planning and accurate shape estimation to enable safe and reliable motion.

### 5.1. Linearized and Discretized Model for Configuration Refinement

Defining the state variables of the target continuum robot system as $x=(p_{2,x},p_{2,y},p_{2,z},$$p_{\mathrm{mid},x},p_{\mathrm{mid},y},p_{\mathrm{mid},z}{)}^{T}\in R^{6}$ and the system inputs as ${\dot{q}}_{A}={\left[{\dot{q}}_{1,1},{\dot{q}}_{1,2},{\dot{q}}_{2,1},{\dot{q}}_{2,2},{\dot{z}}_{T}\right]}^{T}\in R^{5}$, its instantaneous kinematics can be expressed in the following general form:

(33) $$\dot{x}\left(t\right)=g\left(x\left(t\right),{\dot{q}}_{A}\left(t\right)\right)=J_{q}\cdot {\dot{q}}_{A}$$

(34) $$J_{q}=\left[\begin{array}{c} \frac{\partial f_{w,c}\left(\cdot \right)}{\partial {\dot{q}}_{A}^{\left(1\right)}},\frac{\partial f_{w,c}\left(\cdot \right)}{\partial {\dot{q}}_{A}^{\left(2\right)}},\ldots ,\frac{\partial f_{w,c}\left(\cdot \right)}{\partial {\dot{q}}_{A}^{\left(5\right)}}\\ \frac{\partial h_{w,c}\left(\cdot \right)}{\partial {\dot{q}}_{A}^{\left(1\right)}},\frac{\partial h_{w,c}\left(\cdot \right)}{\partial {\dot{q}}_{A}^{\left(2\right)}},\ldots ,\frac{\partial h_{w,c}\left(\cdot \right)}{\partial {\dot{q}}_{A}^{\left(5\right)}} \end{array}\right]\in R^{6\times 5}$$

where $p_{2,x}$, $p_{2,y}$, and $p_{2,z}$ represent the coordinate components of the manipulator’s tip; ${\dot{q}}_{A}^{\left(i\right)}$ denotes the *i*-th component of the input vector ${\dot{q}}_{A}$; and $f_{w,c}\left(\cdot \right)$ and $h_{w,c}\left(\cdot \right)$ respectively correspond to the functional mappings given in (2) and (5).

By applying a Taylor series expansion to (33), the original nonlinear system is linearized and transformed into the following linear time-varying (LTV) system:

(35) $$\begin{array}{cc} \dot{x}\left(t\right) & ={\left.\frac{\partial g}{\partial x}\right|}_{x\left(t\right),{\dot{q}}_{A}\left(t\right)}x\left(t\right)+{\left.\frac{\partial g}{\partial {\dot{q}}_{A}}\right|}_{x\left(t\right),{\dot{q}}_{A}\left(t\right)}{\dot{q}}_{A}\left(t\right)+\omega \left(t\right)\\ \; & =A_{t}x\left(t\right)+B_{t}{\dot{q}}_{A}\left(t\right)+\omega \left(t\right) \end{array}$$

Following the forward Euler method, (35) is discretized, leading to the following discretized linearized model:

(36) $$x\left(k+1\right)=A_{k}x\left(k\right)+B_{k}{\dot{q}}_{A}\left(k\right)+\omega \left(k\right)$$

where $A_{k}={\left.\frac{\partial g}{\partial x}\right|}_{x\left(t\right),{\dot{q}}_{A}\left(t\right)}\cdot T+I_{6}$, $B_{k}={\left.\frac{\partial g}{\partial {\dot{q}}_{A}}\right|}_{x\left(t\right),{\dot{q}}_{A}\left(t\right)}\cdot T$. *T* is the time duration of a single control step, and ω(k) denotes a disturbance term that includes modeling errors.

### 5.2. Design of the Online Configuration Refiner

The objective of the designed configuration refiner is to safely guide the continuum manipulator toward a specified target position while avoiding collisions, guided by the offline-planned configuration sequence. The manipulator’s configuration can be fully characterized by its tip and mid-positions. Accordingly, the cost function for the online configuration refiner is designed as

(37) $$\begin{array}{cc} \; & \min J=\sum_{i=1}^{N_{p}}{∥x\left(k+i|k\right)-x_{\mathrm{ref}}\left(k+i|k\right)∥}_{\underline{\underline{Q}}}^{2}+\sum_{i=1}^{N_{c}-1}{∥\Delta {\dot{q}}_{A}\left(k+i|k\right)∥}_{R}^{2}\\ \; & \;+k_{\mathrm{obs}\;}\sum_{i=1}^{N_{p}}\sum_{j=1}^{N_{\mathrm{obs}\;}}\frac{1}{\rho_{\min\;,j}\left({\dot{q}}_{A}\left(k+i|k\right)\right)+\varepsilon }\\ \; & \;s.t.\;{\dot{q}}_{A}\in \left[{\dot{q}}_{A,\;\min\;},{\dot{q}}_{A,\;\max\;}\right] \end{array}$$

where $x_{\mathrm{ref}}\in R^{6}$ denotes the offline-planned reference configuration, defined by the trajectories of the manipulator’s tip and mid-positions. The term ${∥x\left(k+i|k\right)-x_{\mathrm{ref}}\left(k+i|k\right)∥}_{Q}^{2}$ quantifies the deviation between the actual configuration and the offline-planned reference, which guides both the tip and the mid-positions around their pre-planned trajectories. $Q\in R^{6\times 6}$ and $R\in R^{5\times 5}$ denote the weighting matrices associated with the configuration tracking error and input effort, respectively. The tracking of the mid-segment position can be appropriately relaxed via *Q* to ensure overall motion feasibility. $\rho_{\min}\left(\cdot \right)$ is the distance from the current configuration to the nearest constraint region. $k_{\mathrm{obs}}$ is the weighting coefficient for the collision avoidance penalty term. $N_{p}$ and $N_{c}$ are the prediction horizon and control horizon, respectively.

By substituting (36) into (37), the following reformulation is obtained:

(38) $$\begin{array}{cc} \; & \min\Delta {\dot{q}}_{A}^{T}H\Delta {\dot{q}}_{A}+2f^{T}\Delta {\dot{q}}_{A}+\underset{P_{\mathrm{obs}\;}\left({\dot{q}}_{A}\right)}{\underset{︸}{k_{\mathrm{obs}\;}\sum_{i=1}^{N_{p}}\sum_{j=1}^{N_{\mathrm{obs}\;}}\frac{1}{\rho_{\min\;,j}^{2}\left({\dot{q}}_{A}\left(k+i|k\right)\right)+\varepsilon }}}\\ \; & \;s.t.\;{\dot{q}}_{A}\in \left[{\dot{q}}_{A,\min},{\dot{q}}_{A,\max}\right] \end{array}$$

where $P_{\mathrm{obs}}\left({\dot{q}}_{A}\right)$ is the nonlinear collision penalty term; $H=\Theta^{T}\bar{Q}\Theta +\bar{R}$ is a positive definite matrix that governs the variation in control inputs; and $f=2{\left(x-x_{\mathrm{ref}}\right)}^{T}\bar{Q}\Theta$, with Θ being the control-to-state influence matrix, which describes the cumulative effect of control inputs on future states.

To efficiently solve the nonlinear optimization problem incorporating collision penalty terms (38), the sequential quadratic programming (SQP) framework is adopted, wherein the original problem is iteratively solved via a series of quadratic programming (QP) subproblems.

The gradient of the collision penalty term is given by the chain rule as

(39) $$\nabla P_{obs}\left({\dot{q}}_{A}\right)=\sum_{i=1}^{N_{p}}{\left(\frac{\partial x_{k+i}}{\partial {\dot{q}}_{A}}\right)}^{T}\frac{\partial P_{obs}}{\partial x_{k+i}}$$

For a single prediction step, the gradient with respect to the state vector is given by

(40) $$\frac{\partial P_{\mathrm{obs}}}{\partial x_{k+i}}=\sum_{j=1}^{N_{\mathrm{obs}}}\frac{-2\rho_{\min,j}\left({\dot{q}}_{A}\left(k+i|k\right)\right)\cdot v_{k+i}}{{\left(\rho_{\min,j}^{2}\left({\dot{q}}_{A}\left(k+i|k\right)\right)+\varepsilon \right)}^{2}},\;i=1,\ldots ,N_{p}$$

(41) $$v_{k+i}=\frac{P_{\mathrm{close}\;}-P_{\mathrm{obs}\;,j}}{\rho_{\min\;,j}\left({\dot{q}}_{A}\left(k+i|k\right)\right)}$$

where $P_{\mathrm{close}\;}$ denotes the closest position of the continuum manipulator to constraint regions, determined based on the real-time shape estimation results and the surrounding environment. By linearizing (38) at the current configuration and substituting (39) into it, the QP subproblem is then reconstructed as

(42) $$\begin{array}{cc} \; & \min\Delta {\dot{q}}_{A}^{T}H\Delta {\dot{q}}_{A}+{\left(2f+k_{\mathrm{obs}}\nabla P_{\mathrm{obs}}\left({\dot{q}}_{A}\right)\right)}^{T}\Delta {\dot{q}}_{A}\\ \; & \;s.t.\;{\dot{q}}_{A}\in \left[{\dot{q}}_{A,\min},{\dot{q}}_{A,\max}\right] \end{array}$$

The SQP algorithm solves the original problem (37) iteratively via a series of QP subproblems (42). The detailed steps of the algorithm were provided in [31,32]. Then, a series of control inputs can be obtained:

(43) $$\Delta {\dot{q}}_{A}=\left[\Delta {\dot{q}}_{A}\left(k\right),\Delta {\dot{q}}_{A}\left(k+1\right),\ldots ,\Delta {\dot{q}}_{A}\left(k+N_{c}-1\right)\right]$$

By applying ${\dot{q}}_{A}=\Delta {\dot{q}}_{A}\left(k\right)+{\dot{q}}_{A,\mathrm{ref}}$ to the actual system, the refined configuration for the next time step can be obtained.

## 6. Experiments and Results

### 6.1. Structure and Platform

**Figure 6:**
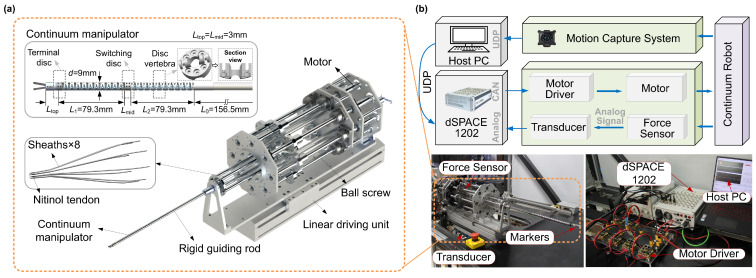
Overall experimental setup: (**a**) structure and parameters of the continuum robot; (**b**) schematic of the experimental platform.

The configuration and parameters of the continuum robot are illustrated in Figure 6a. The system comprises a tendon-driven continuum manipulator and a driving module. The manipulator consists of 42 serially connected vertebrae, with each adjacent pair capable of rotating about two orthogonal directions. It is divided into two flexible segments separated by a switching disc. Each segment provides pitch and yaw motion, while an additional linear actuator enables axial extension, resulting in a five-degree-of-freedom (5-DOF) robotic system. The overall experimental platform is shown in Figure 6b. The system was actuated by nine DC motors (MAXON, DCX 19/26, Sachseln, Switzerland) through ball-screw transmission mechanisms. A tension sensor (DaySensor, DYMH-113, Bengbu, China) was mounted at the end of each driving cable to calibrate the initial zero-state tension. Two passive optical markers were fixed at the distal end of the manipulator, and their real-time position and direction were captured and fed back by a motion capture system (NOKOV, Mars2H, Beijing, China). All algorithms were implemented on a computer (Intel, Ultra 5 225H 1.70 GHz, Santa Clara, CA, USA). Control commands were transmitted via user datagram protocol (UDP) to the dSPACE system (dSPACE, 1202, Paderborn, Germany) for protocol conversion, and then forwarded to drivers (MOTION G, UF01A/03A, Shenzhen, China) to execute the corresponding actuation.

### 6.2. Performance Evaluation and Comparison in Simulated Environments

#### 6.2.1. Effectiveness Validation of CoDE for Configuration Planning

To validate the effectiveness of the CoDE for offline manipulator configuration planning, performance tests were conducted and compared with results based on standard DE. In the experiment, the start and end positions in the operational workspace were set to $P_{ST}=\left(48.5,-65,292.8\right)$ and $P_{ED}=\left(98.5,81.2,232\right)$, respectively. The parameter settings for the CoDE algorithm are listed in Table 1. For comparison, the DE algorithm used a population size twice that of Np, while all other parameters were kept identical.

**Figure 7:**
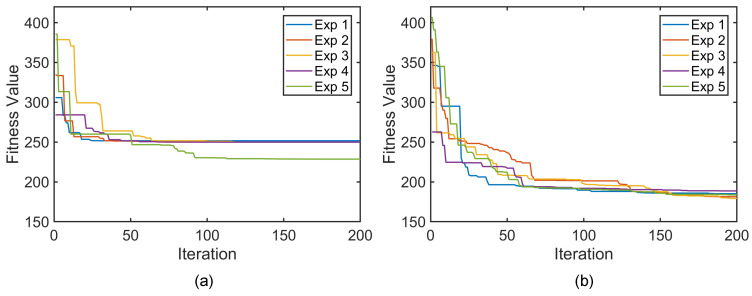
Convergence curves from five independent runs: (**a**) standard DE; (**b**) CoDE.

Five repeated experiments were conducted under identical conditions. The convergence curves of the algorithms are illustrated in Figure 7, and the corresponding quantitative results are summarized in Table 2. The results indicate that the standard DE algorithm tends to converge to local optima; across the five trials, the best fitness value obtained was 228.6. This behavior stems from the difficulty of the DE in effectively exploring multimodal solution spaces in high-dimensional nonlinear optimization problems. In contrast, CoDE exhibited superior fitness values in all trials, with an average fitness of 183.6 over five experiments, representing an improvement of approximately 25.3% compared with DE for this configuration planning task. These results indicate the effectiveness of CoDE for continuum manipulator configuration planning and its improved performance over conventional DE, which is mainly attributed to its co-evolutionary decomposition and inter-population information exchange mechanisms.

#### 6.2.2. Validation and Comparative Analysis in Constrained Environments

**Figure 8:**
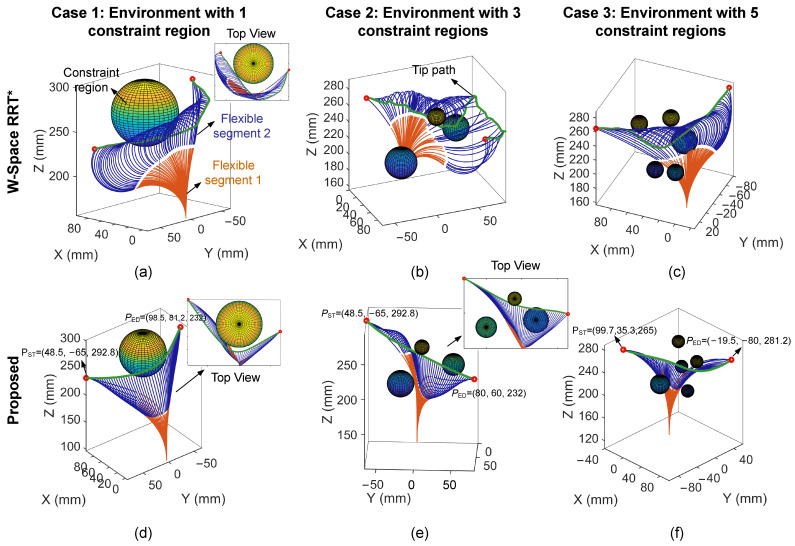
Configuration planning results and comparison across different test cases: (**a**–**c**) results under the W-Space RRT* method; (**d**–**f**) results under the proposed CoDE-based method.

Furthermore, several constrained environments with different complexity were constructed within the robot’s effective workspace to further evaluate the performance of the proposed CoDE-based configuration planning method. The specific environment settings are shown in Figure 8.

RRT-based algorithms have been widely adopted for continuum robot motion planning and have demonstrated good adaptability. To further assess the performance benefits of the proposed approach, the workspace-based RRT* (W-Space RRT*) algorithm was introduced for comparison. Unlike the conventional configuration space-based RRT* (C-Space RRT*), W-Space RRT* reduces unnecessary motions induced by the nonlinear mapping between configuration space and workspace, thereby producing higher-quality configuration trajectories.

The planning results are shown in Figure 8 and Table 3. A smoothness metric is introduced to quantify the trajectory continuity, characterized by the standard deviation (Std) of the trajectory curvature, which is calculated as follows:

(44) $$\gamma =\sqrt{\frac{\sum_{i=1}^{N}{\left(c_{i}-\bar{c}\right)}^{2}}{N}}$$

where $c_{i}$ denotes the curvature at the current position.

As shown in Figure 8, both methods achieve effective collision avoidance within a finite time in the three test environments. However, the tip path generated by the comparative method exhibits noticeable curvature discontinuities, which are primarily attributed to the inherent stochasticity of the sampling process. In contrast, the overall configuration and end-effector trajectory under the proposed CoDE-based method maintain better smoothness, which is expected to facilitate more stable motion in practical operation. To quantitatively evaluate the overall performance of the tip path obtained by configuration planning, a performance metric κ is introduced in Table 3, defined as the ratio of the inverse path length to the computation time. A higher value of κ indicates better overall performance in terms of both path length and computational efficiency. In the relatively simple single-constraint test scenario (Case 1), the W-Space RRT* obtained a solution with comparable path length at a lower computational time. Nevertheless, the proposed CoDE-based method demonstrated a consistent advantage in terms of motion smoothness. Furthermore, in complex and constrained environments (Cases 2 and 3), CoDE exhibited more pronounced performance benefits. Specifically, in Case 2, the CoDE configuration planner generated a shorter tip path with more uniformly distributed curvature. In Case 3, despite a slight increase in computational time, CoDE achieved improvements of 14.7% in the final path length and 96.8% in the smoothness metric γ, respectively. Moreover, as shown in Figure 8d,f, the configuration sequence obtained by the CoDE method satisfies collision-free motion constraints while exhibiting more continuous and natural bending deformations. Throughout the entire motion process, the manipulator moves without excessive body twisting. Such deformation can be achieved with lower tension of driving tendons, which typically implies lower vertebrae friction and reduced uncertainty, thereby allowing the planned configurations to be relatively accurately reproduced in real environments.

### 6.3. Performance Evaluation and Comparison in Real-World Environments

Based on the offline configuration planning solutions, the online refinement mechanism uses real-time shape estimation results to monitor the manipulator–environment interaction for configuration adjustment. Validation and comparison experiments were conducted to further validate the effectiveness of the proposed hybrid framework for collision-free configuration planning.

#### 6.3.1. Effectiveness and Accuracy Evaluation of Shape Estimation

**Figure 9:**
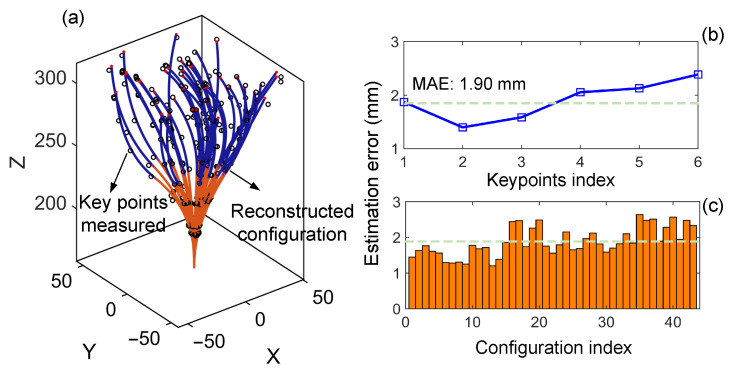
Shape estimation error results of the continuum manipulator: (**a**) estimation and reconstruction results under different configurations, (**b**) distribution of estimation errors at different measurement positions, and (**c**) estimation errors for different configurations.

The accuracy of the UKF-based shape estimation strategy was evaluated to demonstrate its capability to effectively reflect the manipulator’s configuration within constrained spaces, thereby providing prior information for online configuration refinement. Six observation points were evenly selected along the manipulator, from the base to the tip (denoted by black circles in Figure 9a). These positions were measured in real time using a motion capture system. The average estimation error across these six points was used to assess the accuracy of the shape estimation [33]. The parameters were set as $Q_{\mathrm{UKF}}=10^{-3}\times I_{4}$, $R_{\mathrm{UKF}}=10^{-3}\times \mathrm{diag}\left(27,27,27,0.025,0.025,0.025\right)$, $\alpha_{\mathrm{UKF}}=0.01$, and $\beta_{\mathrm{UKF}}=2$.

**Figure 10:**
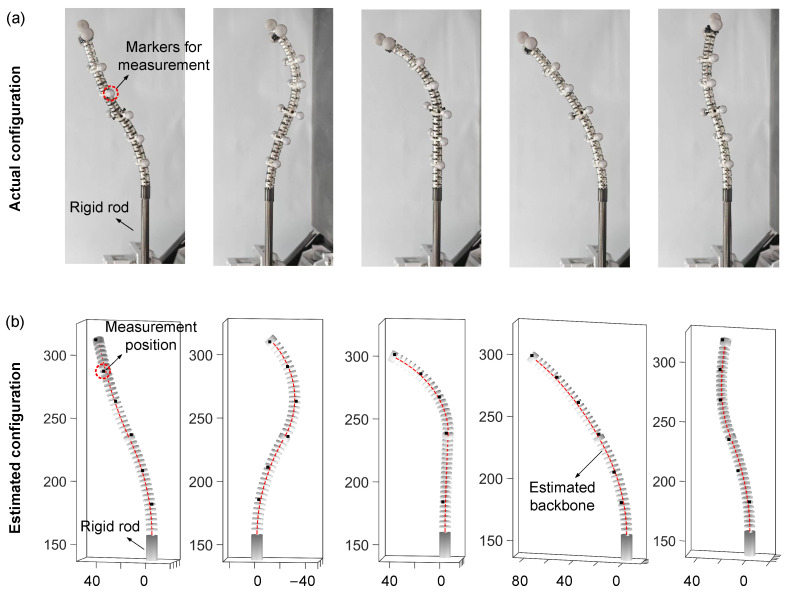
Comparison of actual and estimated shapes for five representative actuated configurations: (**a**) actual shapes; (**b**) estimated shapes.

A total of 43 distinct configurations within the effective workspace were tested. The corresponding experimental results are illustrated in in Figure 9a. The orange and blue curves represent the reconstructed backbone shapes of the continuum manipulator obtained through the UKF-based method, while the black hollow circles denote the observation points measured by the motion capture system. Figure 10 compares the actual shapes of the continuum manipulator under five representative actuation configurations with the corresponding estimates obtained using the algorithm in this paper. Specifically, Figure 10b shows the estimated manipulator shape, where the black square markers denote key positions measured by the motion capture system. It can be observed that the estimated central backbone closely matches the actual manipulator shape. Moreover, all measured key points are located in close proximity to the estimated centerline, demonstrating good agreement between the estimated shape and the ground truth. To further quantify the performance, Figure 9c presents the error distribution over all 43 target test configurations, with detailed error metrics summarized in Table 4. The experimental results show that the proposed method achieves a minimum estimation error of 1.20 mm and a maximum error of 2.64 mm. The mean absolute error (MAE) and root mean square error (RMSE) are 1.90 mm and 1.94 mm, respectively, corresponding to just 1.18% of the total manipulator length, which demonstrates the satisfactory shape estimation accuracy of the proposed approach. Moreover, the close proximity of the MAE to the RMSE indicates that the estimation errors are consistently distributed without significant outliers, reflecting stable performance across different configurations. Furthermore, the average estimation errors at different observation points were statistically analyzed, as shown in Figure 9b. The horizontal axis represents the index of observation points ordered from the base to the tip of the manipulator, and the vertical axis indicates the average estimation error at each point. The results show a slight increasing trend along the manipulator, with all errors remaining within the range of 1.9±0.6 mm. These results demonstrate the reliable shape estimation provided for online configuration refinement and collision avoidance.

Compared with the work in [33,34], the proposed UKF-based method achieves accurate estimation performance while avoiding reliance on extensive sensor feedback on the manipulator. The overall shape of the continuum manipulator is reconstructed using only the tip pose feedback and motor-side encoder position measurements. The encoders are integrated into the actuation system and are mechanically decoupled from the manipulator body, thus imposing no additional space requirements on the arm. Furthermore, unlike the online optimization-based shape estimation approaches in [29,35], the UKF-based framework does not rely on complex iterative computation, which enables real-time shape estimation. In the current setup, tip pose feedback is obtained by capturing two markers on the end effector using a motion capture system (calibration accuracy: 0.52 mm). This configuration can be further simplified by using a 5- or 6-DOF electromagnetic (EM) tracker (Northern Digital Inc., Waterloo, ON, Canada) which provides a nominal positioning accuracy of approximately 0.45 mm [36].

#### 6.3.2. Real-Time Computational Performance Evaluation

**Figure 11:**
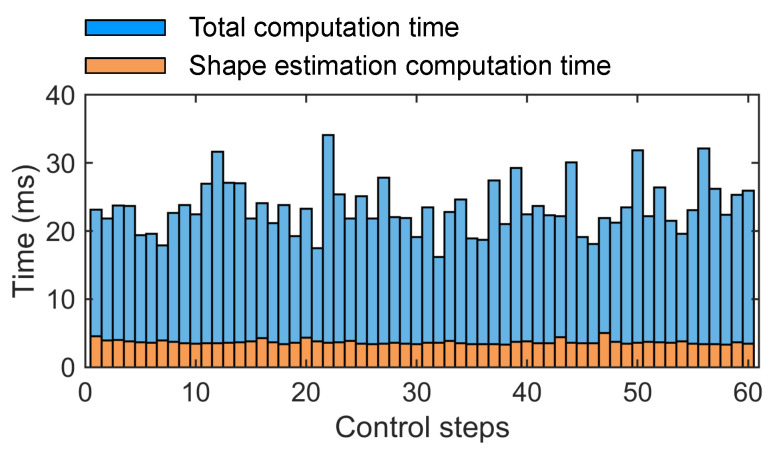
Computation time distribution across 60 consecutive control cycles.

To assess the real-time performance of the proposed method, the computation time within a single control cycle was measured. Figure 11 shows the distribution of computation time over 60 consecutive control cycles, where the blue and orange areas represent the total computation time and the time consumed by the shape estimation module, respectively. The results show an average computation time of 25.8 ms per cycle, with the UKF-based shape estimation accounting for 3.7 ms on average. Under the preset control frequency of 25 Hz (i.e., a 40 ms control cycle), all computational tasks were completed within this time frame, demonstrating the real-time capability of the proposed approach.

#### 6.3.3. Validation and Comparison of Collision Avoidance in Constrained Environments

To further evaluate the performance of the method, collision avoidance experiments were conducted in a real environment. The setup was consistent with Case 3 in Section 6.2, which included five spherical constraint regions. The parameters of the online refiner were set as $N_{p}=10$, Q=diag(2,2,2,0.8,0.8,0.8), R=20×diag(1,1,1,1,0.3), and $k_{\mathrm{obs}}=9\times 10^{4}$. For comparison, the proposed method without online configuration refinement and the W-Space RRT* method were also tested. All experiments were performed under identical environmental constraints.

**Figure 12:**
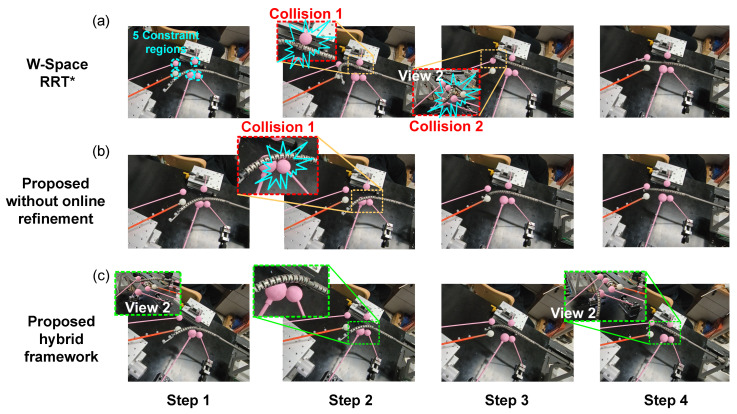
Motion snapshots in constrained environments under different methods: (**a**) W-Space RRT*, (**b**) proposed method without online refinement, and (**c**) proposed hybrid planning framework.

**Figure 13:**
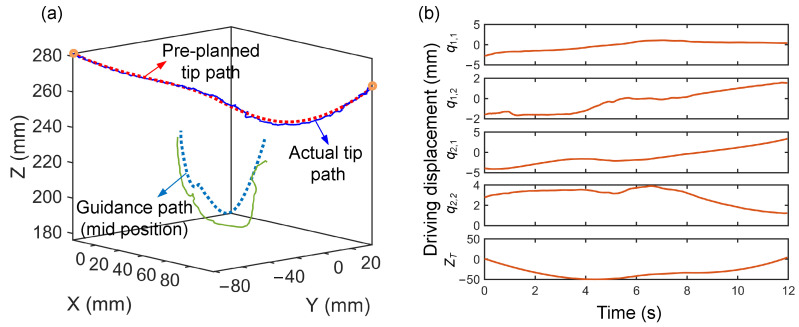
Manipulator motion and driving signals: (**a**) trajectories of the tip and mid segments; (**b**) actuation displacements.

Figure 12 shows the motion processes under the three methods, and the corresponding collision avoidance results are summarized in Table 5. Due to uncertainties caused by friction, the configuration planning results are difficult to accurately reproduce in real environments. This is particularly evident when the manipulator undergoes excessive deformation, as the large tendon tension required further amplifies the deviations, thus leading to collisions in practice (Figure 12a). As illustrated in Step 2 of Figure 12a, the continuum manipulator experiences significant deformation, causing its actual configuration to deviate substantially from the theoretically planned collision-free shape, and thus, resulting in collisions with the nearby obstacle-constrained region. Although, in theory, this issue can be mitigated by expanding the size of the constraint region during planning, this further reduces the limited available space for configuration planning. As shown in Figure 12b, the moderate deformation of the manipulator reduces collisions during motion; however, unsafe movements still persist. In contrast, the proposed hybrid planning framework achieves reliable collision avoidance across the entire constrained workspace (Figure 12c). This effectiveness benefits from accurate shape estimation, which allows the planner to continuously monitor the distance to surrounding obstacles and perform online refinements, thereby enabling reliable collision-free motion of the robotic system. As shown in Figure 13, the red and light blue dashed lines denote the offline-planned paths of the tip and mid-positions, respectively, while the blue and green solid lines represent their actual motions. To ensure reliable collision avoidance during the online re-planning process, the tracking constraint of the mid-position is relaxed by reducing the corresponding weighting term in matrix *Q*. This relaxation allows appropriate deviation of the mid-segment, providing sufficient configuration flexibility for local refinements around the reference configurations, and thus leveraging the global insight of the offline plan to achieve robust collision avoidance under constrained environments.

#### 6.3.4. Performance Comparison Under Different Configuration Adjustment Mechanisms

To further validate the performance advantage of the hybrid configuration planning framework, a null-space-projection-based (NSP-based) collision avoidance approach was selected for comparison, which has been widely used for real-time collision avoidance in constrained environments [21,22,37,38]. Its core idea is to guide the manipulator’s tip along a given trajectory while exploiting the Jacobian null space to perform online adjustment of the manipulator configuration, thereby achieving whole-body collision avoidance. The experimental setup is identical to that in the previous section, and the core collision avoidance potential field law is as follows [21]:

(45) $$U_{r,k}=\left\{\begin{array}{cc} \frac{k_{r}}{\gamma }{\left(\frac{1}{d_{k}\left(q_{A}\right)}-\frac{1}{d_{l}}\right)}^{\gamma } & d_{k}\left(q_{A}\right)\leq d_{l}\\ 0 & d_{k}\left(q_{A}\right)>d_{l} \end{array}\right.$$

where $k_{r}$ and γ are gain coefficients, $d_{k}\left(\cdot \right)$ denotes the sensed minimum distance between the manipulator and the obstacle, and $d_{l}$ is the distance threshold for activating collision avoidance. In this experiment, the algorithm parameters were carefully tuned to $k_{r}=200$, γ=2, and $d_{l}=30$.

**Figure 14:**
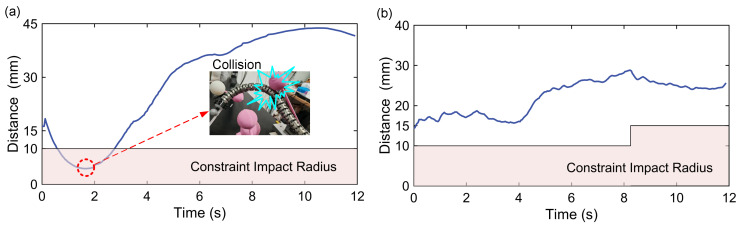
Minimum distance curve between the manipulator and the constraint center during motion: (**a**) NSP-based method; (**b**) proposed method.

Figure 14 shows the distance profile between the continuum manipulator and the center of the constrained region under two methods, where the pink area denotes the influence radius of the nearest constraint. As observed in Figure 14a, for the NSP-based method, portions of the curve enter the constraint influence area during the collision avoidance motion, resulting in a collision with the spherical constraint, as illustrated by the corresponding snapshot. In contrast, the proposed hybrid framework achieves safe collision avoidance in this multiple-obstacle-constrained space, which is primarily attributed to its whole-body configuration-based guidance mechanism. Trajectory-guided NSP planning methods for continuum manipulators perform well in simple constraint spaces with isolated obstacles [21,22]. However, in cluttered environments with multiple obstacles, the inherently myopic nature of the online planner regarding the manipulator configuration increases the risk of being trapped in locally infeasible regions, where no admissible collision avoidance solution can be found given the current configuration. The proposed method exploits the global perspective provided by offline configuration planning. By dynamically sensing the distance between the manipulator and constraint regions, it guides and incrementally refines the whole-body configuration in real time. This combination of offline configuration guidance and real-time whole configuration refinement enables stable collision avoidance in confined spaces with multiple constraints.

## 7. Conclusions

This paper presents a hybrid offline–online configuration planning framework for safe motion of continuum robots in constrained environments. First, a CoDE-based configuration pre-planning method is developed, in which each flexible segment of the manipulator is treated as an independently evolving sub-population to decompose the high-dimensional nonlinear optimization problem, while elite solution sharing enhances global exploration in the search space. Then, a UKF-based shape estimation strategy is constructed to reconstruct the manipulator shape in real time using tip pose feedback, which enables effective safe distance monitoring with a limited number of manipulator-integrated sensors. Experimental results show that the MAE of this estimation is 1.9 mm, accounting for approximately 1.15% of the total manipulator length, which provides a reliable basis for collision avoidance. Finally, guided by the global perspective provided by offline planning, the online refiner performs local refinements of the manipulator configuration by solving an SQP problem around the reference configurations. Unlike conventional tip trajectory-guided configuration planning frameworks, the proposed method leverages a globally optimized whole-body configuration perspective to guide online refinement. The configuration is incrementally adjusted according to real-time shape estimation, enabling reliable collision avoidance in constrained environments. Runtime analysis shows that the average online computation time per control cycle is approximately 25.8 ms, which supports real-time operation on the robotic system with a 25 Hz control frequency. Experiments in multiple constrained environments show its performance advantage in continuum robot configuration planning, and real-world tests further demonstrate its effective and robust collision avoidance performance.

Future work will focus on extending the method to a more flexible three-segment continuum surgical robot and integrating a 5-DOF magnetic navigation sensor at the end-effector, with the aim of exploring potential applications in confined surgical environments.

## Figures and Tables

**Table 1:** Parameter settings for CoDE.

Parameter Name	Value
Population size	Np=20
Scaling factor	F=1
Crossover probability	CR=0.8
Iteration number	iter=150
Weighting coefficient	$\omega_{\eta }=10$, $\omega_{a}=5$
Penalty term	P=50

**Table 2:** Performance evaluation of algorithms under five trials.

Methods	Best Fitness	Worst Fitness	Average	RMS
DE	228.6	251.6	245.9	246.2
CoDE	179.3	188.6	183.6	183.6

RMS: Root mean square.

**Table 3:** Comparison of configuration planning results.

Environment Settings	Method	Tip Path Length	Curvature Std γ	Computation Time	Metric $\kappa \times 10^{-4}$
Case 1	W-Space RRT*	210.8 mm	$2.5\times 10^{-2}$	11.2 s	4.23
	Proposed	201.9 mm	$2\times 10^{-3}$	18.4 s	2.69
Case 2	W-Space RRT*	249.8 mm	$7.6\times 10^{-2}$	42.3 s	0.95
	Proposed	170.4 mm	$5\times 10^{-4}$	27.2 s	2.16
Case 3	W-Space RRT*	207.3 mm	$3.1\times 10^{-2}$	33.1 s	1.46
	Proposed	176.9 mm	$1\times 10^{-3}$	37.5 s	1.51

**Table 4:** Shape estimation error results.

Error Metrics	Value
MinE (mm)	1.20
MaxE (mm)	2.64
MAE (mm)	1.90
RMSE (mm)	1.94
RMSE/$L_{m}$	1.18%

MinE: minimum error; MaxE: maximum error; $L_{m}$: total length of continuum manipulator.

**Table 5:** Comparison of collision avoidance results under different methods.

Methods	Collision Status	Number of Interference Zones
W-Space RRT*	Collided	2
Proposed without refinement	Collided	1
Proposed hybrid framework	Safe	0

## Data Availability

The data presented in this study are available upon request from the corresponding author.

## Abbreviations

The following abbreviations are used in this manuscript:

CoDE	Co-evolutionary improved DE
C-Space	Configuration space
DE	Differential evolution
DOF	Degree of freedom
IK-based	Inverse-kinematics-based
MAE	Mean absolute error
MPC	Model predictive control
NSP	Null space projection
PSO	Particle Swarm Optimization
QP	Quadratic programming
RMSE	Root mean square error
RRT	Rapidly exploring random tree
SQP	Sequential quadratic programming
UDP	User datagram protocol
UKF	Unscented Kalman filter
W-Space RRT*	Workspace-based RRT*
